# Hedgehog Signaling, a Critical Pathway Governing the Development and Progression of Hepatocellular Carcinoma

**DOI:** 10.3390/cells10010123

**Published:** 2021-01-11

**Authors:** Jia Ding, Hui-Yan Li, Li Zhang, Yuan Zhou, Jian Wu

**Affiliations:** 1 Department of Gastroenterology, Shanghai Jing’an District Central Hospital, Fudan University, Shanghai 200040, China; jiading12@fudan.edu.cn; 2 Department of Medical Microbiology and Parasitology, MOE/NHC Key Laboratory of Medical Molecular Virology, School of Basic Medical Sciences, Shanghai Medical College, Fudan University, Shanghai 200032, China; 17111010064@fudan.edu.cn (H.-Y.L.); 19111010054@fudan.edu.cn (L.Z.); 20211010043@fudan.edu.cn (Y.Z.); 3 Department of Gastroenterology & Hepatology, Zhongshan Hospital of Fudan University, Shanghai 200032, China; 4 Shanghai Institute of Liver Diseases, Shanghai Medical College, Fudan University, Shanghai 200032, China

**Keywords:** hedgehog signaling, GLI1/2, hepatocellular carcinoma, cancer-initiating cells, drug resistance, metastasis, Sonic hedgehog ligand

## Abstract

Hedgehog (Hh) signaling is a classic morphogen in controlling embryonic development and tissue repairing. Aberrant activation of Hh signaling has been well documented in liver cancer, including hepatoblastoma, hepatocellular carcinoma (HCC) and cholangiocarcinoma. The present review aims to update the current understanding on how abnormal Hh signaling molecules modulate initiation, progression, drug resistance and metastasis of HCC. The latest relevant literature was reviewed with our recent findings to provide an overview regarding the molecular interplay and clinical relevance of the Hh signaling in HCC management. Hh signaling molecules are involved in the transformation of pre-carcinogenic lesions to malignant features in chronic liver injury, such as nonalcoholic steatohepatitis. Activation of GLI target genes, such as ABCC1 and TAP1, is responsible for drug resistance in hepatoma cells, with a CD133^−^/EpCAM^−^ surface molecular profile, and GLI1 and truncated GLI1 account for the metastatic feature of the hepatoma cells, with upregulation of matrix metalloproteinases. A novel bioassay for the Sonic Hh ligand in tissue specimens may assist HCC diagnosis with negative α-fetoprotein and predict early microvascular invasion. In-depth exploration of the Hh signaling deepens our understanding of its molecular modulation in HCC initiation, drug sensitivity and metastasis, and guides precise management of HCC on an individual basis.

## 1. Introduction

Hedgehog (Hh) signaling is a classic and conserved pathway, controlling embryonic development and tissue repairing under physiological conditions [1]. Disruption of Hh signaling results in an array of developmental disorders in the face, ranging from minor alterations to more serious conditions, such as severe cleft of the lip and palate [2]. Aberrant activation of this pathway has been well documented in malignant transformation, progression, drug resistance and metastatic processes in a number of solid tumors, including glioblastoma, base cell carcinoma, hepatoblastoma, hepatocellular carcinoma (HCC), lung cancer, breast and prostate cancer [3,4,5,6,7]. The unique signaling molecules, i.e., the Sonic (SHh), Desert (DHh) Indian (IHh) ligands, as well as receptor patched (PTCH1), the transmembrane intermediate molecule smoothened (SMO), and transcription factors (GLI1,2,3) are highly expressed in most malignant tissues, and have been considered as biomarkers for progression and prognosis [8,9]. More specifically, SMO has been considered as a useful molecular target for intervention with small molecules, such as LDE225 and GDC-0449, in suppressing this pathway [10,11]. SMO inhibitors, vismodegib (GDC-0449), sonidegib (NVP-LDE225) and saridegib (IPI-926), have been approved by the FDA for the treatment of glioblastoma and base cell carcinoma [12,13] or for clinical trials in the treatment of pancreatic, breast and colon cancer [14]. However, the efficacy of SMO inhibitors in other solid carcinoma may not be as effective as in glioblastoma or base cell carcinoma due to activation of the non-canonical Hh signaling pathway or other oncogenic signaling pathways [15]. The application of these inhibitors and development of other candidates are an active area of molecular intervention in cancer therapy. 

At the same time, molecular targeting agents and checkpoint inhibitors are optimal selections for refractory malignancies, such as pulmonary, hepatocellular and breast carcinoma, and primary and induced resistance to these therapeutics prompts basic and clinical investigations on the molecular interplay underlying drug resistance. Hh signaling has been found to be one of key signaling pathways in modulating drug resistance through ATP-binding cassette (ABC) transporters, such as ABCC1 and TAP1, in our previous studies [16,17]. 

Intrahepatic and extrahepatic invasion and distal metastasis are the main cause of death in HCC. Involvement of the Hh signaling in every step of progressive behaviors has been gradually elucidated at molecular levels and with clinical relevance. As one of the clinically important parameters, circulating tumor cells (CTCs) are the source of new metastatic lesions [18]. Their cancer stem cell-like features, surface markers, as well as signaling molecules in controlling intravasation, survival in the bloodstream, and formation of new lesions are crucial topics in cancer biology. The involvement of Hh signaling in these processes is intriguing to determine how HCC metastasis happens with multiple intrinsic and environmental factors working in a coordinating fashion. 

Most methodologies in detection of the Hh signaling molecules use molecular approaches, such as RT-PCR, for the mRNA levels, and Western blot or immunohistochemical staining for the protein expression levels and location in tissues. So far, quantitative determination of Hh signaling molecules has not been used to monitor drug sensitivity or prognosis prediction, although a sensitive ELISA kit is available for the Sonic Hh ligand (SHh) measurement [7]. Since Hh ligands are secreted in an autocrine and paracrine mechanism from tumor cells or mesenchymal cells in the tumor, there is potential application of their assay in tumor tissue or body fluid for clinical measurement. Thus, it is clinically relevant to develop sensitive and reliable assays for prompt, high-throughput application in clinical services. 

The present review aims to provide an overview covering the signaling mechanism, modulation and interaction with other signaling molecules, such as TGF-β. The major attention is focused on the molecular mechanism of Hh signaling in modulating drug resistance and metastasis of HCC. Additionally, a novel bioengineering assay for the detection of SHh in HCC specimens and its potential application are elaborated. The use of pharmacotherapeutic candidates for intervention of Hh signaling in cancer therapy has been covered recently by extensive reviews [14,15,19,20], and thus we did not have this topic as a key focus of this review. The outline of the present review is highlighted in the bullet points.

- Hedgehog (Hh) signaling is aberrantly activated from chronic inflammation to malignant transformation.
- Targeting Hh molecules, such as SMO or GLI, confers rational intervention for malignancies.
- Hh participates in drug resistance by maintaining stem-like properties, onset of EMT and induction of ABC transporters in HCC.
- Hh signaling contributes to HCC metastasis by mediating EMT, MMP production or involvement in multiple steps of metastasis.
- A sensitive aptasensor-based assay was developed to detect Sonic Hh ligand in HCC specimens with acceptable positivity and high specificity.

## 2. Hh Signaling in Transformation to Malignant Cells

### 2.1. Aberrant Activation of Hh Signaling in Normal Liver and HCC 

Hh signaling is activated via canonical or non-canonical pathways under different conditions. The canonical Hh pathway includes secretory ligands (Sonic, Indian and Desert), the 12-pass transmembrane protein patched1 (PTCH1), heptahelical transmembrane G-protein-coupled receptor smoothened (SMO) and transcription factor GLI proteins (GLI1, GLI2 and GLI3) [21]. In the absence of ligands, PTCH1 inhibits the activity of SMO, which in turn recruits suppressor of fused (SUFU) to inactivate the GLI proteins [22]. With the inhibitory interaction of SUFU and being phosphorylated by protein kinase A (PKA), GLI2/3 proteins undergo partial proteolysis and act as transcriptional repressors (GLI2/3R) [23,24]. The binding of Hh ligands to PTCH1 liberates SMO in the primary cilia [25,26], leading to suppression of PKA enzymatic activity and translocation of full-length GLI2/3 into the nucleus as transcription activators [27,28]. As an exclusive activator, GLI1 is regulated by GLI2/3 at the transcriptional level [29,30].

As mentioned above, Hh signaling in adult livers is normally silent. Hh ligands are expressed in injured hepatocytes, activated hepatic stellate cells (HSCs), Kupffer cells, endothelial cells, progenitor cells and natural killer cells; and activated Hh signaling is involved in tissue repair [31]. During an embryonic stage, Hh signaling is transiently expressed in hepatoblasts, and is inhibited when they matured to hepatocytes, indicating it is essential for differentiation and maturation of liver progenitor cells [32]. In adult liver, the Hh signaling is maintained at a low level, may participate in the maintenance of progenitor cell pool, hepatocellular zone difference as well as lipid hemostasis [31]. As will be discussed more extensively in a later section, Hh signaling is activated in a variety of chronic liver injuries, such as alcoholism, steatosis, cholestasis or viral infection, and is responsible for hepatocellular generation, HSC activation and other processes [31]. 

Detection of Hh molecules in HCC specimens indicates that the reactivation of Hh signaling contributes to hepatic carcinogenesis. SHh ligand was detected in 60% HCC tissues, especially in the tumor nests [33,34]. Increased expression of SMO and GLI1 was directly correlated to a larger tumor size [35]. Both PTCH1 and GLI1 were observed in more than 50% of tumor tissues, and a higher ratio of GLI1 mRNA in HCC/noncancerous liver was significantly correlated with recurrence and short overall survival (OS) [36,37]. Immunoreactivity of GLI2 was presented in parenchymal cells of 84.6% HCC cases, and nuclear GLI2 staining was significantly correlated with a poor differentiation status as well as portal vein tumor thrombosis [38]. 

For canonical Hh signaling, primary cilia function as a signaling hub for SMO-dependent GLI activation. The non-canonical Hh signaling pathway does not involve the ligand and receptor (PCTH1) and is operated in a SMO-independent fashion. The non-canonical pathways of GLI activation were observed in hepatoma or cholangiocarcinoma cells with impaired cilia expression [39]. The process of Hh signaling activation in malignant cells lacking primary cilia remains unclear. As a member of cyclic AMP-regulated kinase families, protein kinase C (PKC) was reported to phosphorylate serine or threonine residues of the GLI transcription factors; however, it displayed less specificity than PKA [40,41]. The isoform, PKC-δ, decreased GLI1 transcriptional activity and its nuclear translocation in Hep3B cells; whereas, lacking of PKC-δ contributed to disrupt Hh signaling response, which was consistent with the finding that PKC-δ expression was undetectable in HCC specimens with Hh activation [42]. Transforming growth factor-β (TGF-β) plays dual roles (suppressive or tumorigenic) in HCC under different conditions [43]. A direct induction of GLI2 protein expression by TGF-β1 was observed in dermal fibroblasts, keratinocytes and breast carcinoma cells in an SMO-independent manner [44]. Canonical and non-canonical Hh signaling pathways mediated by Sonic ligands or TGF-β1 co-exist in hepatoma cells and HCC specimens [8,45]. Our unpublished data suggest that GLI2 functions as a downstream target of TGF-β/Smad3 in poorly differentiated hepatoma cells (Cancer Res 2020; 80 (16 Supplement): DOI: 10.1158/1538-7445.AM2020-6336). Different mechanisms in Hh signaling activation may be attributed to the heterogeneity of HCC, and Hh molecules, including SMO and GLIs, are considered to be targets for molecular intervention, such as GDC-0449 and LDE225 for SMO, and arsenic trioxide (ATO) or GANT61 for GLIs [46].

### 2.2. The Effect of Hh Signaling on Pro-Tumorigenic Transformation

Hh signaling not only orchestrates embryogenesis but also regulates regeneration and inflammatory response to liver damage. Liver epithelial (hepatocyte or cholangiocyte) cells are induced to secrete Hh ligands under pathologic conditions, and in turn activate Hh signaling in neighboring Hh-responsive cells via a paracrine mechanism. Initial GLI2 activation upregulates the transcription of GLI1 and the entire signaling pathway. Persisting Hh signaling activation in injured hepatocytes and non-parenchymal cells contributes to hepatocellular carcinogenesis under pathologic conditions, such as nonalcoholic steatohepatitis (NASH) with significant fibrosis [31].

Chronic infection with hepatitis B virus (HBV) and hepatitis C virus (HCV) increased hepatic expression of the Sonic and Indian ligands, which bound to the PTCH1 receptor on myofibroblast-like HSCs and hepatic sinusoidal endothelial cells (SECs) to initiate liver fibrogenesis and angiogenesis [47]. Not only as an inducer of SHh, PTCH1 and GLI2 production, HBV X protein (HBx) directly interacted with GLI1 to increase transactivation of the Hh target genes [48]. HBx transgenic mice gradually developed steatosis, dysplastic nodules and visible HCC by 12 months, accompanied by hepatic expression of SHh, IHh, PTCH1 and GLI2. Blockage of PTCH1 and SMO activity with vismodegib-inhibited tumor growth in both HBx-transgenic mice and HBx-positive human HCC xenograft models [46]. These studies suggested that HBV-mediated HCC is dependent on Sonic Hh signaling activation.

**Figure 1:**
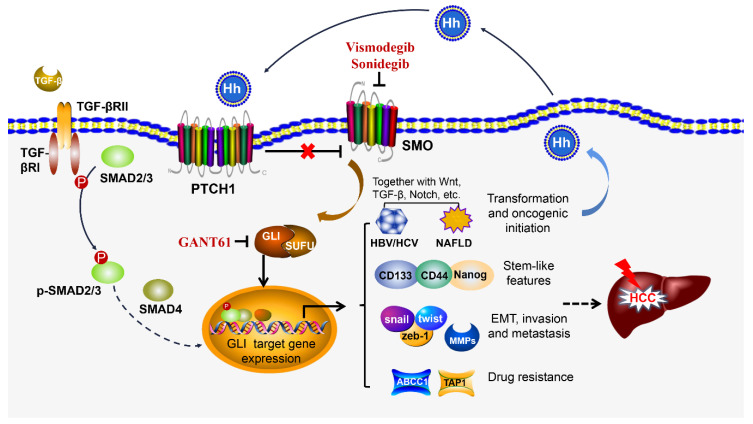
Hedgehog (Hh) signaling pathway contributes to hepatic carcinogenesis. The canonical Hh pathway starts from the Hh ligands binding to the 12-pass transmembrane protein patched 1 (PTCH1), which liberates SMO and further promotes the translocation of GLI into the nucleus. The non-canonical Hh pathway does not involve the ligand and receptor (PCTH1), and is operated in an SMO-independent fashion. In fact, it is activated by other factors, such as TGF-β. TGF-β binds to TGF-β receptor II and facilitates the phosphorylation of SMAD2/3, and then p-SMAD2/3 is translocated into the nucleus together with SMAD4. The GLI transcription factor may interact with the SMAD complex and co-transact GLI targeting genes although how SMAD and GLIs work together in co-transactivation of the targeting genes is unclear. These targeting genes are involved in chronic inflammation and its transformation to oncogenic initiation, together with the fibrogenic (TGF-β) and oncogenic (c-Myc, Wnt, Nanog) factors. Hh signaling plays a critical role in maintenance of stem-like properties (CD133, CD44 and Nanog) in onset of epithelial mesenchymal transition (EMT) and in HCC invasion and metastasis through Snail, Twist, Zeb-1 and MMPs, as well as in drug resistance through ABCC1 and TAP1. The Hh signaling is self-amplified through a positive feedback of its signaling molecules as targeting genes (PTCH1 and GLI1), and the activated cells produce more ligands under an oncogenic status.

In nonalcoholic fatty liver disease (NAFLD), the Hh activity is positively correlated with the severity of liver damage. Lipotoxicity-injured hepatocytes with ballooning degeneration were the main source of Hh ligand production and accumulation of GLI2-positive Hh-responsive cells paralleled with the liver fibrosis stage in NAFLD patients [49,50]. The Hh-responsive cells include liver progenitor cells, hepatocytes, HSCs and liver macrophages. Hh activation increased osteopontin production by hepatocytes and NKT cells [51,52] drove quiescent HSCs to trans-differentiate into activated myofibroblasts [53], recruited macrophages to secrete proinflammatory cytokines and eventually aggravated steatohepatitis and fibrosis [54]. Prolonged hepatic SHh expression in a transgenic mouse model resulted in hepatocellular apoptosis, HSC activation, accumulation of extracellular matrix (ECM) components and fibrogenesis [55]. Although a liver tumor failed to be induced in this persisting SHh expression model, findings from a high-fat diet-induced fatty liver mouse model demonstrated that tumors were observed in obese mice after administration of a carcinogenic agent, diethylnitrosamine (DEN). In the latter model, steatotic hepatocytes primarily secreted IHh, which was necessary for HCC development. DEN failed to induce HCC in lean mice, and obese IHh^fl/fl^ knockout (KO) mice displayed a decreased tumor burden. Hh-responsive HSCs and EpCAM^+^ ductal cells upregulated MYC and TGF-β2 expression and were transformed into fibrotic and pro-tumorigenic phenotypes [56]. With growing evidence, it is speculating that excessive accumulation of SHh/IHh ligands in hepatocytes and Hh-responsive cells within steatotic liver accelerates the transition from NAFLD to fibrosis and further transformation to HCC. Other oncogenic factors, such as c-Myc, Wnt, Nanog and Hippo signaling molecules, may also be involved in this complicated transformation [57,58]. Figure 1 schematically illustrates the modulating role of Hh signaling in oncogenic initiation, progression, drug resistance and metastasis of HCC.

Hepatocellular adenoma (HCA) is a kind of benign tumor; however, it has an increased risk of malignant transformation when telomerase reverse transcriptase (TERT) was activated [59,60]. A new molecular subgroup of HCA was characterized by SHh activation due to fusion of the promoter of GLI1 and inhibin-βE. HCAs bearing with Hh activation were associated with obesity and a higher rate of hemorrhage, and 6% of patients were identified to have HCC in this subgroup [61]. A transgenic mouse model demonstrated that persisting SHh expression enhanced HCA initiation induced by P53^R172H^ and KRAS^G12D^ mutation, suggesting that Hh signaling activation facilitates malignant transformation in combination with other oncogenes, such as c-Myc and Wnt signaling [55]. 

## 3. Hh Signaling in Drug Resistance 

Clinical management of HCC needs to be balanced with tumor burden, liver reserve function and patients’ general health. According to the Barcelona Clinic Liver Cancer (BCLC) system, resection, liver transplantation and ablation are recommended to patients with a solitary nodule ≤ 2 cm or less than three nodules ≤ 3 cm (BCLC stage 0 and A); chemoembolization is recommended to patients with multinodular (BCLC stage B); and systemic therapies are recommended to patients with macrovascular invasion or extrahepatic spread (BCLC stage C) [62]. Less than 40% of HCC patients are suitable for curative treatments when diagnosed at early stages. Even with resection, recurrence reaches 70% in 5 years [63]. Although an increasing number of therapeutic agents have been approved for advanced HCC treatment, including the first line sorafenib and lenvatinib and second line regorafenib, cabozantinib and ramucirumab, overall survival of HCC patients has not been remarkably improved. One of the reasons for this failure is attributed to patients with advanced HCC developing primary and secondary resistance to these molecular targeting medications or checkpoint inhibitors [64]. Multiple Hh target genes, including stem cell markers (CD44 and CD133), epithelial–mesenchymal transition (EMT) transcription factors and drug resistance-associated ABC proteins, contribute to drug resistance in the treatment of HCC [65].

### 3.1. Maintenance of Cancer Stem-Like Properties 

In an adult liver, bilineage-potent hepatic progenitor cells (HPC) are located within the canals of Hering, and its appearance is in parallel with the extent of the ductular reaction driven by hepatocellular damage [66,67]. The HPCs co-express stem surface markers (EpCAM, NCAM, CD133 and CD44), endodermal transcription factors (SOX9 and SOX17), hepatocellular (albumin and CK18) and cholangiocytic (CK7/19) markers, and Hh ligands (Sonic and Indian) [68]. However, persistent Hh signaling activation during extensive liver injury is thought to initiate carcinogenesis and maintain a subpopulation with stemness. Activation of Hh was observed in both CD44^+^ and CD133^+^ organoids or cells derived from HCC patients. HCC-derived CD44^+^ organoids displayed sorafenib-resistance, and a GLI inhibitor, GANT61, suppressed proliferation of both CD44^+^-HCC patient-derived organoids and CD44^+^ hepatoma cells, indicating that CD44^+^ cells with a sorafenib-resistant phenotype was maintained by the Hh signaling [69]. A long non-coding RNA associated with HDAC2 (lncHDAC2) was identified in CD13^−^/CD133^+^ cells derived from primary HCC specimens, and was reported to suppress PTCH1 expression and consequently promote self-renewal of tumor-initiating cells through Hh signaling [70]. Sestrin-3 belongs to a multifunctional protein family that participates in protecting against antioxidative stress and metabolic homeostasis [71,72]. Sestrin-3 directly interacted with GLI2 to inhibit its nuclear translocation, and sestrin-3 deficiency contributed to hepatic carcinogenesis partially by Hh signaling activation. HCC patients with less sestrin-3 expression had a tendency of worse survival, and sestrin-3 knock-out (KO) mice demonstrated a higher tumor burden and expression of stem markers (CD133 and CD44) compared to the wild-type group in a DEN combined with a choline-deficient high-fat diet (CD-HFD) model [73]. In summary, a growing body of evidence suggests that Hh signaling participates in the maintenance and renewal of liver progenitor cells under a physiologic condition and may contribute to the differentiation and maturation of hepatocellular and/or cholangiocytic lineages under chronic liver injury. These progenitor cells could be the cell sources for malignant transformation under a pathogenic condition; for instance, extensive fibrosis, activation and participation of Hh signaling were well documented in a NASH-neoplastic model in our previous studies and clinical specimens [49,58].

### 3.2. Induction of Epithelial–Mesenchymal Transition (EMT)

Epithelial–mesenchymal transition (EMT) denotes a process by which epithelial cells lose their polarity and adhesion, acquire cell mobility and transform to the mesenchymal phenotype [74]. This transformation is mediated by several transcription factors, including Twist, Snail and Zeb-1. Loss of E-cadherin, an epithelial-specific adhesion molecule and expression of mesenchymal markers, such as vimentin, indicate the presence of EMT. EMT occurs during embryonic development, wound healing response and neoplasia [75]. In the latter case, hepatoma cells with EMT characteristics were more resistant to sorafenib, and patients with a scirrhous type of HCC were associated with early disease recurrence [76]. EMT transcription factors (Twist, Snail and Zeb-1) were reported as the target genes of Hh signaling in poorly differentiated hepatoma cells, and Hh transactivation endowed the poorly differentiated hepatoma cells with a mesenchymal phenotype and chemo-resistance [6,77]. As a major EMT-promoting cytokine, TGF-β is usually upregulated in fibrotic/cirrhotic livers and was found to be dysregulated in 40% of HCCs. Sustained presence of TGF-β induced the combined activation of the SHh and Wnt signaling pathways during the EMT process in HCC with a mesenchymal phenotype [78]. EMT conveyed HCC cells to gain the ability to escape from death in a hypoxic microenvironment. Hypoxia-inducible transcription factor-1 (HIF-1) promoted GLI1 activation and led to an onset of EMT and invasiveness of the hepatoma cells [8]. Chondroitin sulfate synthase-1 (CHSY-1) is an enzyme that synthesizes chondroitin sulfate, which is abundant in both the ECM and cell surface of the tumor [79]. CHSY-1 enhanced SHh binding to the cell surface of hepatoma cells, co-localized with chondroitin sulfate, and promoted pulmonary metastasis in a mouse xenograft model. HCC patients with a high expression of CHSY-1 were associated with a worse histologic grade and lower survival rate [80]. In summary, as a regulator for EMT, Hh signaling regulates progressive behaviors, drug resistance and metastatic properties through activation of EMT transcription factors, such as Snail, Twist and Zeb-1. TGF-β1 may be one of the critical cytokines involved in activation of Hh signaling in a non-canonical way, in which the SMAD3/4 complex may interact with GLI2 for transactivation of the target genes without the ligand–receptor interaction and SMO involvement [81]. 

### 3.3. Mediation of ABC Transporters

The ATP-binding cassette (ABC) transporter superfamily functions in transporting sugars, peptide, ions and drugs. ABCB1, ABCC1 and ABCG2 are three of the most characterized ABC transporters involved in multiple drug resistance through increasing the efflux of the chemotherapeutic agents [82,83]. Of note, ABCB2 and ABCF1 were reported to play a role in chemoresistance of pancreatic ductal adenocarcinoma and HCC [84,85]. As a marker of hepatic progenitor cells, ABCB1 was observed in canalicular staining of normal liver, whereas ABCC1 was negative [86]. Similar to ABCB1, ABCG2 was identified as a stem cell marker to maintain a side population exhibiting cancer stem cell-like properties in HCC [87]. Immunohistochemical staining of ABCG2 in normal liver was observed in the canals of Hering, bile ducts and some periportal hepatocytes [88]. Increasing evidence has demonstrated that ABCB1 and ABCG2 were direct target genes of GLI1 in hematological malignancies and solid tumors, and their expression was inversely correlated with response to chemotherapy [89,90,91,92]. However, the correlation between expression of ABC transporters and prognosis remains controversial in HCC. Several studies suggested that ABCB1 or ABCG2 overexpression was an independent factor for predicting a shorter survival time [93,94,95,96], whereas another study reported that ABCB1 expression had no association with aggressive pathologic features and survival [97]. This discrepancy may be explained by two reasons: (i) polymorphism of ABCB1 and/or ABCG2 determined the clinical response to sorafenib in patients with HCC [98]; and (ii) expression of the ABC transporters was associated with the differentiation degree of the HCC. ABCB1 and ABCG2 were mainly expressed in well-differentiated hepatoma cells, whereas poorly differentiated hepatoma cells expressed ABCC1 and ABCB2 (TAP1), accompanied by aberrant activation of the Hh signaling [17]. We have demonstrated that Hh transcription factors GLI1/2 were able to bind to the consensus sequence in the promoter of ABCC1 or ABCB2 to facilitate resistance to sorafenib, doxorubicin and cisplatin in poorly differentiated hepatoma cells. Suppression of GLI1/2 by RNA interference approaches or GANT61, a GLI inhibitor, resulted in a decrease in expression of ABCC1 or ABCB2, and partially restored the chemosensitivity in these poorly differentiated hepatoma cells [16,17]. Moreover, ATO, another GLI1 inhibitor, improved the survival of a spontaneous mouse model of medulloblastoma with activated Hh pathway signaling [99]. In addition, GLI binding sites in the promoter region of six ABC transporters, namely ABCA2, ABCB1, ABCB4, ABCB7, ABCC2 and ABCG1, were confirmed in a recent study, and the inhibition of GLI1 reduced the expression of these ABC transporters in colorectal cancer cells [100]. These studies have further confirmed the governing role of Hh in the mediation of drug resistance in poorly differentiated hepatoma cells, and implied that molecular intervention against the Hh signaling cascade may be a feasible approach in the correction of drug resistance for refractory HCC [17]. 

## 4. Hh Signaling Contributes to Metastasis of HCC

HCC often presents as early microvascular invasion (78.6% (22/28) in resected tissue) [101] and 10–40% with a portal vein tumor thrombus (PVTT) at the time of diagnosis; the occurrence of PVTT indicates the missing of a resectable option although multidisciplinary approaches are available for adjuvant therapy [102,103]. However, the median overall survival without treatment for HCCs with visible PVTT is 2.7–4 months [104]. HCC recurrence of implants was seen in 16% of recipients within 13 months of transplantation [105]. Thus, liver failure due to local invasion and metastasis in other organs are the main causes of death in HCC patients. Meanwhile, mRNA levels of the receptor (PTCH1) and GLI1 were found to be indicative of recurrence after resection [36]. Overall, the SHh signaling is considered to be the target to suppress the progression of this deadly malignancy [106].

### 4.1. Hh-Mediated MMP Production and Onset of EMT Are Essential for Metastasis

The particular feature of liver sinusoids allows easy local invasion of the HCC, and the malignant nodules may lack a connective capsule. Multiple studies have demonstrated that the positivity of Hh signaling molecules, such Sonic ligands, PTCH1 and GLIs, were about 50–70% in HCC specimens, which was much higher than the pericancerous liver tissue of a different base disease background, such as HBV or HCV infection or NAFLD [17,36,106]. The enhanced expression reflected by elevated mRNA and/or protein levels correlated with the advanced stage of the malignant progression, which is often reflected by the Milan or Barcelona staging criteria for HCC. Other molecules involved in the metastatic process, such as MMPs, S100A4 and EMT transcription factors (Snail, Twist, Slug and Zeb-1), were positive in metastatic lesions in other tissue, ascites or distal organs, and may reflect their modulatory role in the early metastatic process, such as intravasation and transporting via the bloodstream or lymphatic vessel network [17]. It is more convincing that the circulating tumor cells (CTCs) from an HCC were found to have a high level of Hh signaling activity. SMO and GLI1 were detected in CTCs from breast cancer patients by immunohistochemistry and RNA in situ hybridization and used as markers of the heterogeneity of the CTCs [107]. At the same time, CTCs express cancer stem cell markers, such as CD90, CD133, EpCAM, CK19 and Nanog, etc. [18,108,109], which supports the concept of circulating cancer stem cells (CSCs) as the cell origin for the wide spreading of HCC in the body [18]. They are important parameters for monitoring relapse, drug efficacy and prognosis in precise medicine practice [109,110]. However, it is unclear how Hh signaling participates in survival or formation of new metastatic lesions in other organs or tissue. 

The metastasis involves a multiple-step process, including invasion, the disruption of the basement membrane by tumor cells, intravasation, transport and extravasation as well as colonization in other tissue [111]. For each of these steps, Hh signaling may take part in a direct or indirect fashion, for instance through EMT, mesenchymal–epithelial transition (MET) or a pre-metastatic niche. The hepatic sinusoids do not have a complete basement membrane, and endothelial cells possess many fenestrae for exchanges of micro-molecules between the lumen of sinusoids and the Disse space. The capillarization of sinusoids reduces the capacity of the nutrient and metabolite exchanges in chronic liver injury with fibrosis and cirrhosis. Pericellular fibrosis is the characteristics of NASH [112], and a thick fibril boundary, especially with the scirrhous type of HCC, may hinder the infiltration of inflammatory or malignant cells. Thus, it is critical for cancer cells to gain the ability to penetrate connective tissue and enter the blood circulation. To achieve this capability, synthesis and secretion of collagenase, such as matrix metalloproteinase1,2,9 (MMP1,2,9) are features of metastatic cells. Our previous study found that CD133^−^/EpCAM^−^ Huh-7 cells, when passing through transwell selection, formed a unique subpopulation, Huh-7-trans, and had extremely high levels of MMP expression compared with their native Huh-7 hepatoma counterpart cells, and at the same time, the GLI1/2 expression levels were accordingly increased. Addition of an SMO inhibitor, LDE225, partially suppressed the expression of MMPs and GLI1/2 and attenuated their invasive behaviors [11], indicating that the metastatic behaviors are under control of the Hh signaling pathway. Moreover, truncated GLI1 was identified in these Huh-7-trans cells, and was suggested to be able to transact MMP gene expression in other studies [113]. Additionally, RNA-Seq data confirmed that these highly metastatic cells exhibited a much elevated expression of S100A4 and Twist1, suggesting that EMT may also participate in their highly metastatic behaviors [11,17]. Our findings are supported by other observations demonstrating that enhanced Hh signaling plays a critical role in the invasive properties of HCC [80]. 

### 4.2. In Situ Model of Liver Xenograft and Metastasis

**Figure 2:**
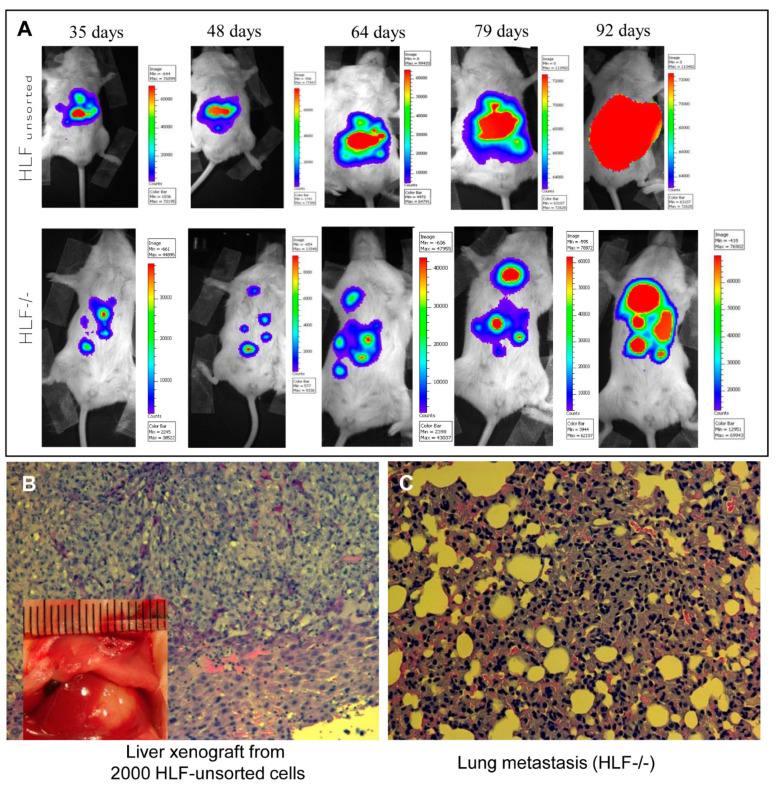
In situ model of a liver xenograft and metastasis. Human hepatoma cells (HLF and HLF CD133^−^/EpCAM^−^) were injected directly into the medium lobe of the liver in NOD-SCID-IL2-γR(-/-) NSG mice under isoflurane anesthesia. These cells were transduced with lentiviral vector LUX-PGK-EGFP before the injection. One month after the injection, the mice were imaged with an IVIS 100 imaging system (Xenogen Corp.) every other week for longitudinal tracking of the formation of xenografts and metastasis over the next two months (**A**). (**B**) An in situ xenograft in the mouse liver was visible with histology confirmation. (**C**) Micrograph of the pulmonary metastatic invasion into alveoli or interstitial tissue (hematoxylin and eosin staining, 200×). The animal experiments were performed with the ethic approval by the institutional committee of animal care and use, and followed the NIH guidelines of experimental animals.

In order to study the metastatic properties of in situ xenografts in immunodeficient mice, we implanted 2000 HLF unsorted or HLF CD133^−^/EpCAM^−^ hepatoma cells in the medium lobe of the liver in NOD-SCID-IL2-γR(-/-) NSG mice. These cells were transduced with a lentiviral vector encoding the firefly luciferase gene (LUX-PGK-EGFP) before injection [114]. As shown in Figure 2, 35 days after the injection, the injected cells were able to grow as xenografts in the liver (HLF) or abdomen (HLF-/-). The luminescent signal became more intense with a bigger area over the next two months in the same mouse injected with HLF cells; whereas, the area of luminescent signal from the mouse injected with HLF-/- cells was diffused with formation of new nodules in both the abdomen and chest (Figure 2A). A new xenograft tumor was found in the liver of the mouse injected with HLF cells (Figure 2B insert) and confirmed by histology. In contrast, the wide spreading of the HLF-/- cell-derived nodules was confirmed in both the abdomen and lungs when the mouse was sacrificed. Figure 2C exhibits the microscopic metastasis of the HLF-/- cells in alveoli or pulmonary interstitial tissue. Therefore, this in situ liver xenograft model with longitudinal tracking of tumor growth and metastasis by a luminescent imaging modality confers a valuable tool to investigate the metastatic feature of specific hepatoma cells (Huh-7-trans or HLF-/-) under different interventions [6,11]. As demonstrated in our previous study, the Hh activity in these cells is highly activated [6], and may be crucial given the highly metastatic characteristic of HLF-/- hepatoma cells. 

### 4.3. Circulating Tumor Cells and Self-Seeding in Implants after Transplantation

It is common for cells to acquire mesenchymal features to be able to migrate from the original locus to distal sites through EMT. The EMT transcription factors (Snail, Twist, Slug and Zeb-1) [115] are often upregulated in solid tumors with hypoxia when they are treated with radiofrequency ablation (RFA) or transarterial chemoembolization (TACE) or in highly metastatic cells, such as Huh-7-trans [8,11]. Enhanced EMT marker expression in resected HCC tissue is associated with a high possibility of recurrence [76]. CTCs are often positive for CSC markers, at the same time, Hh signaling is activated, although other malignant signaling pathways, such as TGF-β, Wnt-β-catenin, Nanog or Stat 3/4, are often positive [107,109]. In other words, the metastatic process may involve multiple signaling pathways with significant heterogeneity. This also confers an explanation for the fact that only a fraction of CTCs is able to survive and form metastatic lesions in other tissues. 

### 4.4. MET and the Microenvironmental Niche for Development of New Lesions

The preferable sites for malignant metastasis depend on the CTCs, blood and lymphatic supply and microenvironmental niche of the tissue. Intrahepatic invasion, portal vein tumor thrombus and abdominal, pulmonary and intracranial metastasis are often seen with HCC. One unique phenomenon is the recurrence of HCC in implants of liver transplantation with the cell origin of the recipient, indicating that malignant cells spreading to other sites of the body re-circulate back to the preferable environment of the implant liver. This self-seeding happened in 5.7% of 60 cases 40 months after transplantation, and may be more often seen in living-donor recipients (31.8%) within 3 years [18,116]. The molecular interplay underlying self-seeding is poorly understood, and one study suggested that inter-cellular communication mediated by exosome-bearing signaling molecules, such as miRNAs, may facilitate this process [117]. However, a familiar and favorable microenvironment (niche) may be more responsible for HCC-derived CTCs to recognize the liver as a seeding site. 

One phenotypic switch from CTCs to return the epithelial feature of HCC is mesenchymal–epithelial transition (MET), which is not yet fully understood regarding self-reseeding in the implants or establishment of new metastatic nodules in other sites. However, the HGF–cMET signaling axis may play a critical role in this process based on the evidence summarized in a recent review [118]. The signaling mechanism coordinates CD46 (c-MET internalization) and TGF-β, EGFR, VEGFR and HER2 for signaling transduction and amplification, and may be well integrated with Hh signaling since TGF-β signaling may act on the non-canonical pathway in activation of the Hh target genes, such as PTCH1, GLI1, BCL2, Snail, Zeb-1/2, etc., in the event of metastasis [81]. TGF-β was found to directly induce GLI2 to promote metastasis of melanoma, glioblastoma, breast and ovarian carcinoma [81]. However, a similar study has not been seen for HCC yet. 

In summary, metastasis is a complex process and involves coordinated action of multiple signaling pathways and is controlled by mutations, CSCs, the microenvironmental niche and other factors. Nevertheless, how Hh signaling participates in this multiple-step process is largely unknown, especially for HCC. It is relatively clear that Hh signaling modulates the synthesis and production of MMPs for assisting early extravascular process and governing EMT through modulating the production of transcription factors (Snail, Twist, Slug and Zeb-1). More studies are warranted to elucidate whether and how Hh signaling controls the survival, formation of metastatic lesions in other tissue with other signaling molecules in a coordinating fashion in a reliable animal model, such as in situ xenograft tumor formation with live imaging of longitudinal tracking. 

## 5. Novel Bioengineering Assay of Hh Signaling Molecules for HCC Detection

Hh signaling molecules, such as ligands (SHh), receptors (PTCH1) or transcription factors (GLI1/2) in the tumor tissue or body fluid, may reflect tumor progression, metastasis, drug sensitivity or treatment response [3,7,33]. Previous investigations, including our data, have supported that SHh is a useful biomarker for tumor metastasis, drug resistance and a target for tumor treatment [6,11,16]. The secretary nature of SHh from tumor cells offers an advantageous opportunity to be detected in cell or tissue lysates or body fluids. However, quantitative determination of Hh signaling molecules has not been used for clinical service, nor as a parameter for monitoring drug sensitivity or prognosis prediction, although a sensitive ELISA kit is available for SHh ligand measurement. Since Hh ligands are secreted in an autocrine and paracrine mechanism, there is potential application of its measurement in tumor tissue or body fluid, such as peripheral blood, ascites, cerebrospinal fluid (CSF) or malignant pleural effusion, and may reflect its leakage from adjacent tissue or organs with neoplastic invasion. Thus, there is an urgent need to develop sensitive and reliable assays for prompt and high throughput application in clinical services. In our recent study, a reliable aptasensor-based assay was successfully developed by conjugating Texas-Red-labeled aptamer (AP32) to microbeads, and was used to analyze the SHh content in hepatoma cell lysates, serum and HCC specimens [101]. The method exhibited a broad detection range from 0.07 to 62.5 nM with a low detection limit of 69 pM, and a recovery rate of 104.6 ± 3.9% in serum. When the assay was used to measure the SHh content in HCC specimen lysates, it possessed a 57.1% positivity and 100% specificity in distinguishing HCC from pericancerous tissues. It was valuable that SHh was above the upper limit in nearly 70% of AFP-negative patients, and by combining both AFP and SHh assays, the positive rate was increased to 85.7%, indicating that SHh has a compensating role for diagnosis of HCC in AFP-negative patients. Moreover, elevated SHh levels indicated portal vein invasion at a 77.8% positive rate. The assay enables a rapid, sensitive and reliable detection of SHh in a variety of sample types, has an acceptable positive rate and superior specificity in distinguishing HCC from pericancerous liver tissue, and may offer a novel bioassay for prediction of early metastasis (such as portal vein invasion) and poor prognosis for HCC patients. The assay may be extended for any other tumors with significant involvement in Hh signaling activity, such as glioblastoma and basal cell carcinoma, for further clinical validation. 

## 6. Perspectives and Conclusions

As one of most refractory malignancies, the death rate of HCC ranks the third, whereas its prevalence lies in the sixth among malignancies. The high death rate is due to its primary or secondary resistance to drug therapy and early metastasis, such as local or portal vein invasion and distal metastasis. In other words, the refractory status and metastasis of HCC account for major causes of lower survival rate. As a classic morphogenic factor, the Hh signaling participates in the malignant transformation in chronic liver injury, oncogenic initiation, progression, drug resistance and metastatic process. The specific ligands, receptors, SMO transmembrane protein and transcription factors (GLI1/2) have become molecular targets for cancer therapy or biomarkers for prognosis prediction. The application of SMO inhibitors, vismodegib, sonidegib or saridegib, has been approved for the treatment of glioblastoma and base cell carcinoma. Clinical trials of these inhibitors in other malignancies, such as pancreatic, lung and colon cancers, are on-going. 

Our previous studies have demonstrated that Hh signaling molecules, such as SHh ligand and GLI1/2, were increased at mRNA and/or protein levels in 50–70% of HCC arisen from HBV-positive patients, which were consistent with reports from other studies. Moreover, high signaling activity has been identified in hepatoma cells with a CD133^−^/EpCAM^−^ surface marker profile, and was associated with EMT, drug resistance and aggressive behaviors. Further studies have uncovered that increased TAP1 and ABCC1 through both GLI1/2 were largely responsible for the primary drug resistance in these CD133^−^/EpCAM^−^ subpopulations. It is promising when GLI1/2, ABCC1 or TAP1 was suppressed, hepatoma cells became more sensitive to chemotherapeutics with a much lower half maximal inhibitory concentration (IC50), indicating the critical role of Hh signaling in modulating the drug sensitivity in these cells. Increasing evidence suggests that Hh signaling modulates the metastatic process through EMT, MMP production and may participate in the mobilization and survival of CTCs. For liver transplant recipients, self-seeding is a pivotal issue for HCC recurrence in transplanted liver, and affects the function of the implant organs and survival of the recipients. Although the literature is scarce regarding whether Hh signaling contributes to the establishment of new metastatic lesions, its oncogenic and promoting action on the microenvironmental niche may explain its role in facilitating the multiple steps of metastasis. 

With multidisciplinary efforts, a sensitive and reliable aptasensor-based bioassay has been established to directly measure SHh ligand in resected HCC specimens. This assay is able to detect SHh at the pmol levels. A pilot study has demonstrated that SHh levels in liver tissue and HCC were in a detectable range, and the measurement could distinguish malignant and pericancerous liver tissue with 100% specificity when the upper limit cut-off was set at a 99% confidence interval. Moreover, the measurement was compensatory for detection of AFP-negative HCC cases and could indicate early microvascular invasion, which is an early sign of portal vein thrombus and poor prognosis. 

In conclusion, upregulated Hh signaling molecules are frequently observed in HCC, and may participate in the initiation, progression, drug resistance and metastasis of the malignancy through EMT or other target genes, such as ABCC1, TAP1 or MMPs. Targeting Hh signaling molecules, SMO and GLI1/2 may confer therapeutic options for improvement of the treatment outcome. The quantitative determination of SHh may offer a sensitive and reliable assay for monitoring drug sensitivity, metastasis or prognosis, although additional clinical investigations in a larger cohort are needed for validation of its broad application.

## Abbreviations

ABCB2/TAP1: ATP-binding cassette subfamily B member/transporter 1; ABCC1: ATP-binding cassette subfamily C member 1; ABCG2: ATP-binding cassette subfamily G member 2; AFP: α-fetoprotein; BCLC: Barcelona Clinic Liver Cancer; CHSY-1, Chondroitin sulfate synthase-1; CSCs: cancer stem cells; CTCs: circulating tumor cells; DEN, diethylnitrosamine; ECM, extracellular matrix; EMT: epithelial mesenchymal transition; GLI1: GLI family zinc finger 1; GLI2: GLI family zinc finger 2; HCC: hepatocellular carcinoma; HCV, hepatitis C virus; Hh, hedgehog; HIF-1, hypoxia-inducible transcription factor-1; HPC, hepatic progenitor cells; HSCs: hepatic stellate cells; IC50: the half maximal inhibitory concentration; MET: mesenchymal epithelial transition; MMP: matrix metalloproteinase; NAFLD: nonalcoholic fatty liver disease; NASH: nonalcoholic steatohepatitis; OS, overall survival; PKA: protein kinase A; PVTT, portal vein tumor thrombus; SECs, sinusoidal endothelial cells; SUFU: suppressor of fused; S100A4: S100 calcium-binding protein A4; TERT, telomerase reverse transcriptase; TGF-β1, transforming growth factor-β1; VEGFR, vascular endothelial growth factor receptor; WNT, Wingless-type MMTV integration site family member.
